# Calprotectin (S100A8/S100A9) detects inflammatory activity in rheumatoid arthritis patients receiving tocilizumab therapy

**DOI:** 10.1186/s13075-022-02887-7

**Published:** 2022-08-19

**Authors:** Michael Gernert, Marc Schmalzing, Hans-Peter Tony, Patrick-Pascal Strunz, Eva Christina Schwaneck, Matthias Fröhlich

**Affiliations:** 1 Department of Medicine II, Rheumatology and Clinical Immunology, University Hospital of Würzburg, Oberdürrbacher Str. 6, 97080 Würzburg, Germany; 2 Asklepios Klinik Altona, Rheumatology and Clinical Immunology, Paul-Ehrlich-Straße 1, 22763 Hamburg, Germany

**Keywords:** Calprotectin, S100A8/S100A9, Rheumatoid arthritis, Tocilizumab, C-reactive protein, Inflammation marker

## Abstract

**Background:**

Assessing serological inflammation is difficult in tocilizumab (TCZ)-treated rheumatoid arthritis (RA) patients, as standard inflammation parameters, like erythrocyte sedimentation rate (ESR) and C-reactive protein (CRP), are influenced by interleukin-6-receptor inhibition. Calprotectin in the serum, also named S100A8/S100A9, might be a more useful inflammation parameter in TCZ-treated patients.

**Methods:**

Sixty-nine RA patients taking TCZ were included. Serum-calprotectin levels were assessed, as well as ESR, CRP, need for a change in disease-modifying anti-rheumatic drugs due to RA activity (= active RA), and the RA clinical disease activity score (CDAI). Forty-five RA patients taking tumor-necrosis factor-inhibitors (TNFi) were investigated for the same parameters.

**Results:**

TCZ-treated patients with active RA had higher calprotectin values than not active RA patients (4155.5 [inter quartile range 1865.3–6068.3] vs 1040.0 [676.0–1638.0] ng/ml, *P* < 0.001). A calprotectin cut-off value of 1916.5 ng/ml resulted in a sensitivity and specificity of 80.0 %, respectively, for the detection of RA disease activity. Calprotectin values correlated with CDAI-scores (*r* = 0.228; *P* = 0.011). ESR and CRP were less suitable to detect RA activity in TCZ-treated patients. Also TNFi-treated patients with active RA had higher calprotectin values compared to not active RA (5422.0 [3749.0–8150.8] vs 1845.0 [832.0–2569.0] ng/ml, *P* < 0.001). The calprotectin value with the best sensitivity and specificity for detecting RA activity was 3690.5 ng/ml among TNFi-treated patients.

**Conclusion:**

Calprotectin in the serum can be a useful inflammation parameter despite TCZ-treatment.

**Supplementary Information:**

The online version contains supplementary material available at 10.1186/s13075-022-02887-7.

## Background

Inflammation parameters in the serum are a useful tool to monitor disease activity in inflammatory rheumatic diseases. In rheumatoid arthritis (RA), mostly erythrocyte sedimentation rate (ESR) and C-reactive protein (CRP) are routinely assessed. CRP and acute phase protein synthesis in the liver is regulated by interleukin-6 (IL-6) [1, 2]. Tocilizumab (TCZ) is one monoclonal antibody inhibiting the IL-6 receptor. With the approval of IL-6 receptor inhibitors for the treatment of RA, diagnostic problems occurred, as the ESR and CRP could no longer reliably display inflammatory activity. Consequently, disease activity scores such as disease activity score (DAS) 28-ESR or DAS28-CRP are also influenced by IL-6 receptor inhibition [3]. The simplified disease activity score (SDAI) also contains the CRP for its calculation [4]. Evaluation of disease activity therefore is more difficult and has to rely on clinical examination, imaging techniques, and patient-reported outcome parameters. Thus, serological markers functioning as inflammation parameters, which are less disturbed by IL-6 receptor inhibition would be helpful.

Calprotectin, a heterodimer of the two calcium-binding proteins S100A8 and S100A9 (synonyms are calgranulin A and B or myeloid related protein [MRP]-8 and MRP-14), is a major cytosolic protein in monocytes and neutrophils, secreted during infections, malignancy, and inflammation [5, 6]. Calprotectin concentrations in the blood increase soon upon exposition to endotoxin [7]. Calprotectin can induce secretion of proinflammatory cytokines in macrophages by upregulation of nuclear factor kappa B in RA [8]. Calprotectin in the serum was shown to be a useful inflammation parameter in other inflammatory diseases comprising polymyalgia rheumatica [9], giant cell arteritis [10], and spondyloarthritis [11]. Measuring fecal calprotectin to date is an established method to determine intestinal activity in inflammatory bowel diseases [12].

The measurement of calprotectin in the serum as inflammation parameter in RA (without IL-6 receptor inhibitors) has been investigated and showed a correlation with radiographic damage scores and standard inflammation parameters [13]. It also showed a positive correlation with synovitis detected with ultrasound [14].

Few data is available for the use of calprotectin in RA with TCZ-treatment. Only one study of 33 patients is published [15], suggesting calprotectin to be helpful. The correlation of calprotectin with disease activity of RA has been described for biological disease-modifying anti-rheumatic drugs (DMARDs) apart from IL-6 receptor inhibitors [16] and was described as more sensitive for disease activity than established acute-phase reactants [17, 18]. We therefore implemented the measurement of calprotectin routinely in our tertiary university center and can present real world data on the use and usefulness of calprotectin in RA patients receiving tocilizumab therapy.

## Patients and methods

### Patients and definitions

One hundred fourteen patients, fulfilling the 2010 ACR/EULAR classification criteria for rheumatoid arthritis [19], were included in this single-center retrospective study. Further inclusion criteria were as follows: Treatment with either TCZ (69 patients with 125 included calprotectin values, as from some patients more than one time point was available) or tumor-necrosis factor-inhibitors (TNFi) (45 patients with 53 calprotectin values) for at least 3 months, availability of calprotectin-, ESR- and CRP value and clinical assessment (including DAS28-ESR, DAS28-CRP, SDAI, and clinical disease activity-index [CDAI]). Exclusion criteria were conditions confounding serological inflammation parameters: Active infectious disease, currently systemic-treated malignancy, pregnancy, and major surgery within the last 4 weeks. Data was collected between the years 2019 to 2022. Data was taken from the patients’ electronic files (EMIL by itc-ms.de, Marburg, Germany and SAP SE, Walldorf, Germany).

The CDAI, comprising the sum of 28 swollen joints, 28 tender joints, patient global, and physician global assessment [20], was assessed in all patients and divided in categories: CDAI-remission ≤ 2.8, low disease activity ≤ 10, moderate disease activity ≤ 22, high disease activity > 22 [21]. Furthermore, the RA patients were grouped in two groups: (1) Not-active patients, who remained on their disease-modifying anti-rheumatic drug (DMARD) without adding additional immunosuppressive medications, and (2) active patients, who received a change or an increase in DMARDs due to inflammatory activity of RA.

### Calprotectin measurement

Serum calprotectin was collected in serum-gel vials (S-Monovette Z-Gel, Sarstedt, Nümbrecht, Germany) and measured with EliA™ Calprotectin 2 wells (wells coated with monoclonal mouse antibodies against calprotectin) with a Phadia™ 250 device (both Thermo Fisher Scientific, Freiburg, Germany). For each measurement cycle, a commercial negative and positive control (Thermo Fisher Scientific) was analyzed. Serum samples were centrifuged within 1h after blood collection and stored between 2 and 8°C until measurement. Stable calprotectin values can be measured with this assay within 7 days [22].

ESR (reference range in the first hour 3–8 mm) and CRP (reference < 0.5 mg/dl) were measured in the central laboratory of the University Hospital of Würzburg. CRP was detected by immunoturbidimetry (Tina-quant C-reactive Protein IV, cobas, Roche, Mannheim, Germany).

### Statistical analysis

Shapiro-Wilk tests were used to test for normal distribution. As normal distribution was mostly absent, medians with interquartile ranges (IQR) were shown. To test for differences between unpaired groups, Mann-Whitney *U* tests were performed for continuous variables and Fisher’s exact tests for categorical variables. Spearman’s tests were used to calculate correlations. Receiver-operator characteristic (ROC) curves were calculated to detect cut-off values with the best sensitivity and specificity. SPSS Statistics v 28.0 (IBM, Armonk, New York) was used for statistical analysis. For data collection Excel (Microsoft, Redmond, Washington) was used. Figures were grouped by using Photoshop (Adobe, San Jose, California). Two-tailed *P-*values less than 0.05 were considered significant.

## Results

### RA patients receiving tocilizumab

#### Characteristics of RA patients receiving tocilizumab

Inflammation parameters were obtained from 69 RA patients. All patients received TCZ for at least 3 months. Forty-eight of 69 (69.6 %) were female; the median age was 64.0 years (range 33–89). Fifty-four of 69 (78.3 %) were rheumatoid factor (RF)-positive; 51/69 (73.9 %) were anti-citrullinated protein antibody (ACPA)-positive. All ACPA-positive patients were RF-positive. Fifty of 69 (72.5 %) received TCZ subcutaneously and 19/69 (27.5 %) intravenously. No concomitant DMARD was present in 43/69 (62.3 %) patients. Thirteen of 69 (18.8 %) received prednisolone (median dosage 5.0 mg daily [inter quartile range, IQR 3.3–5.0]), 12/69 (17.4 %) methotrexate (MTX), and 1/69 (1.4 %) lefunomide (LEF). The median activity parameters of the TCZ-treated patients were as follows: DAS28-ESR 1.5 (IQR 1.1–2.2), DSA28-CRP 1.7 (1.2–2.2), SDAI 5.0 (2.0–8.0), and CDAI 5.0 (2.0–8.0). The median inflammation parameters of the TCZ-treated patients were as follows: ESR in 1h 4.0 (2.0–6.0) mm, CRP 0.0 (0.0–0.1) mg/dl, and calprotectin 1072.0 (686.5–1916.5) ng/ml. Patients’ characteristics are summarized in Table 1.

**Table 1:** Characteristics of the study population

Characteristics	RA patients receiving tocilizumab	RA patients receiving TNFα inhibitors	***P***-value RA patients with tocilizumab vs TNFα inhibitors
Female, *n* (%)	48/69 (69.6)	37/45 (82.2)	0.186
Age, median (range), years	64.0 (33–89)	60.0 (30–81)	0.011*
Disease duration, median (IQR), years	15.0 (9.0–23.8)	13.0 (6.0–23.5)	0.306
Rheumatoid factor positivity, *n* (%)	54/69 (78.3)	32/45 (71.1)	0.505
Anti-citrullinated protein antibody positivity, *n* (%)	51/69 (73.9)	32/45 (71.1)	0.830
*Biological DMARD*			
Tocilizumab subcutaneous, *n* (%)	50/69 (72.5)	na	na
Tocilizumab intravenous, *n* (%)	19/69 (27.5)	na	na
Adalimumab, *n* (%)	na	27/45 (60.0)	na
Etanercept, *n* (%)	na	11/45 (24.4)	na
Certolizumab-pegol, *n* (%)	na	4/45 (8.9)	na
Golimumab, *n* (%)	na	3/45 (6.7)	na
*Concomitant DMARD*			
None, *n* (%)	43/69 (62.3)	12/45 (26.7)	<0.001*
Prednisolone, *n* (%)	13/69 (18.8)	8/45 (17.8)	1.000
Methotrexate, *n* (%)	12/69 (17.4)	30/45 (66.7)	<0.001*
Leflunomide, *n* (%)	1/69 (1.4)	3/45 (6.7)	0.302
*Median disease activity parameters*			
DAS28-ESR, (IQR)	1.5 (1.1–1.2)	2.4 (2.0–3.4)	<0.001*
DAS28-CRP, (IQR)	1.7 (1.2–2.2)	1.9 (1.4–2.6)	0.029*
SDAI, (IQR)	5.0 (2.0–8.0)	5.0 (1.4–8.4)	0.535
CDAI, (IQR)	5.0 (2.0–8.0)	5.0 (1.0–8.2)	0.815
*Median inflammation parameters*			
ESR in 1 h, mm (IQR)	4.0 (2.0–6.0)	16.0 (6.0–24.5)	<0.001*
CRP, mg/dl (IQR)	0.0 (0.0–0.1)	0.1 (0.0–0.5)	<0.001*
Calprotectin, ng/ml (IQR)	1072.0 (686.5–1916.5)	2135.0 (929.5–4072.5)	<0.001*

*CDAI* clinical disease activity index, *CRP* C-reactive protein, *DAS* disease activity score, *DMARD* disease-modifying anti-rheumatic drug, *ESR* erythrocyte sedimentation rate, *IQR* interquartile range, *na* not applicable, *RA* rheumatoid arthritis, *SDAI* simplified disease activity index, *TNF* tumor-necrosis factor. *Significant difference in a Mann-Whitney *U* test or Fisher’s exact test, respectively

#### Calprotectin is increased in tocilizumab-treated active RA

**Fig. 1:**
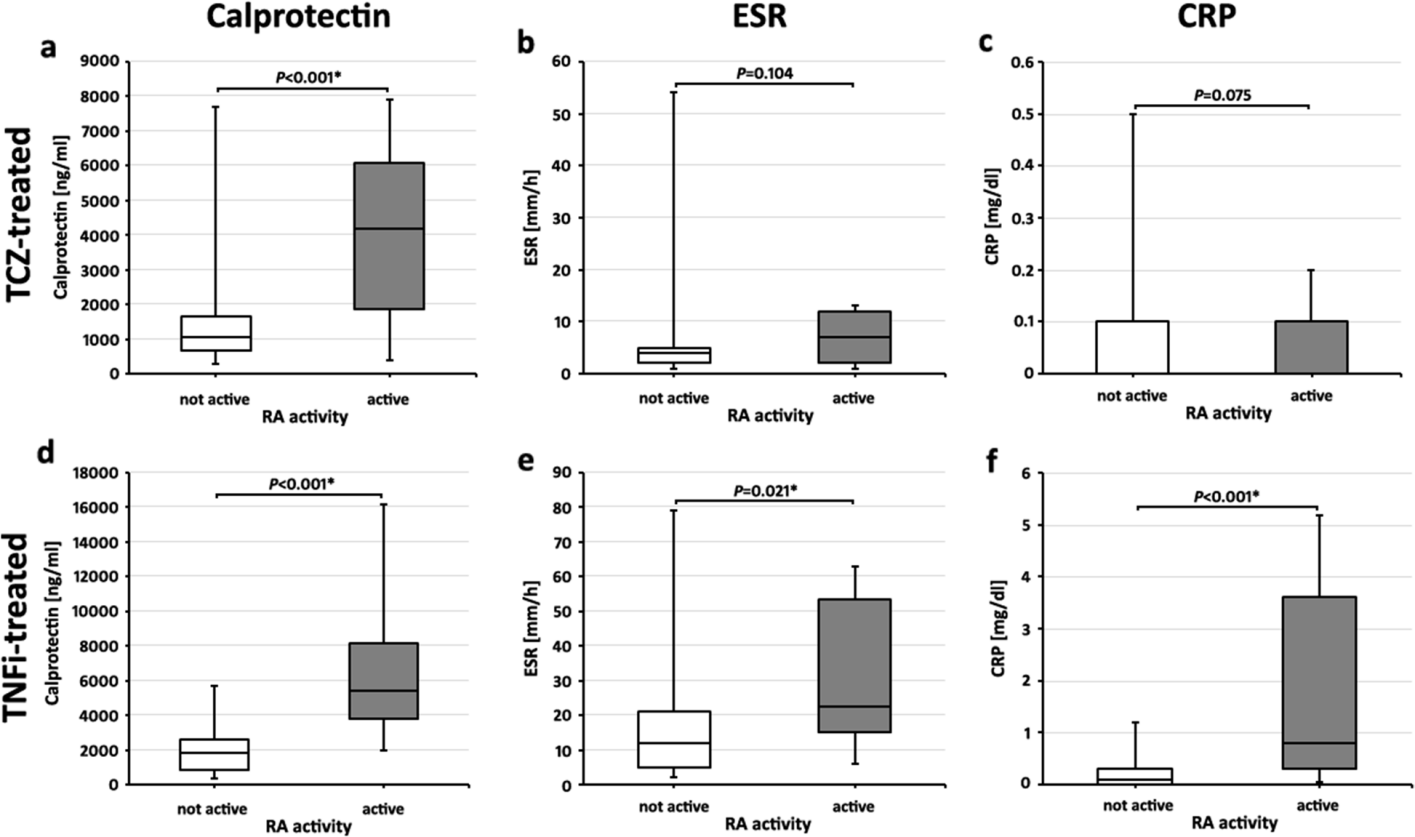
Usefulness of serological inflammation markers in detecting active RA (requiring a change of their disease modifying anti-rheumatic drug, grey box plots) from not active RA (white box plots). Shown are calprotectin (**a**, **d**), ESR (**b**, **e**), and CRP values (**c**, **f**) in tocilizumab (TCZ)-treated RA patients (**a**–**c**) and in tumor necrosis factor alpha-inhibitor (TNFi)-treated RA (**d**–**f**) patients. Boxplots show medians with 25th and 75th percentiles; whiskers indicate minimums and maximums, respectively. *Significant differences in a Mann-Whitney *U* test

The cohort of RA patients receiving TCZ were divided in two groups: (1) one group of patients, who continued their DMARD regimen (i.e. tocilizumab + concomitant medication) (not active), and (2) patients, who needed a change or escalation of their DMARDs due to inflammatory activity of RA (active). One hundred twenty-five calprotectin values were included in this study as from some patients more than one time point was measured. The calprotectin value in the serum was significantly lower in the not active RA patients (*n* = 115) compared to the active patients (*n* = 10) (1040.0 (676.0–1638.0) ng/ml vs 4155.5 [1865.3–6068.3] ng/ml, *P* < 0.001) (Fig. 1a).

#### Calprotectin exhibits good sensitivity and specificity despite tocilizumab treatment in detection of RA activity

**Fig. 2:**
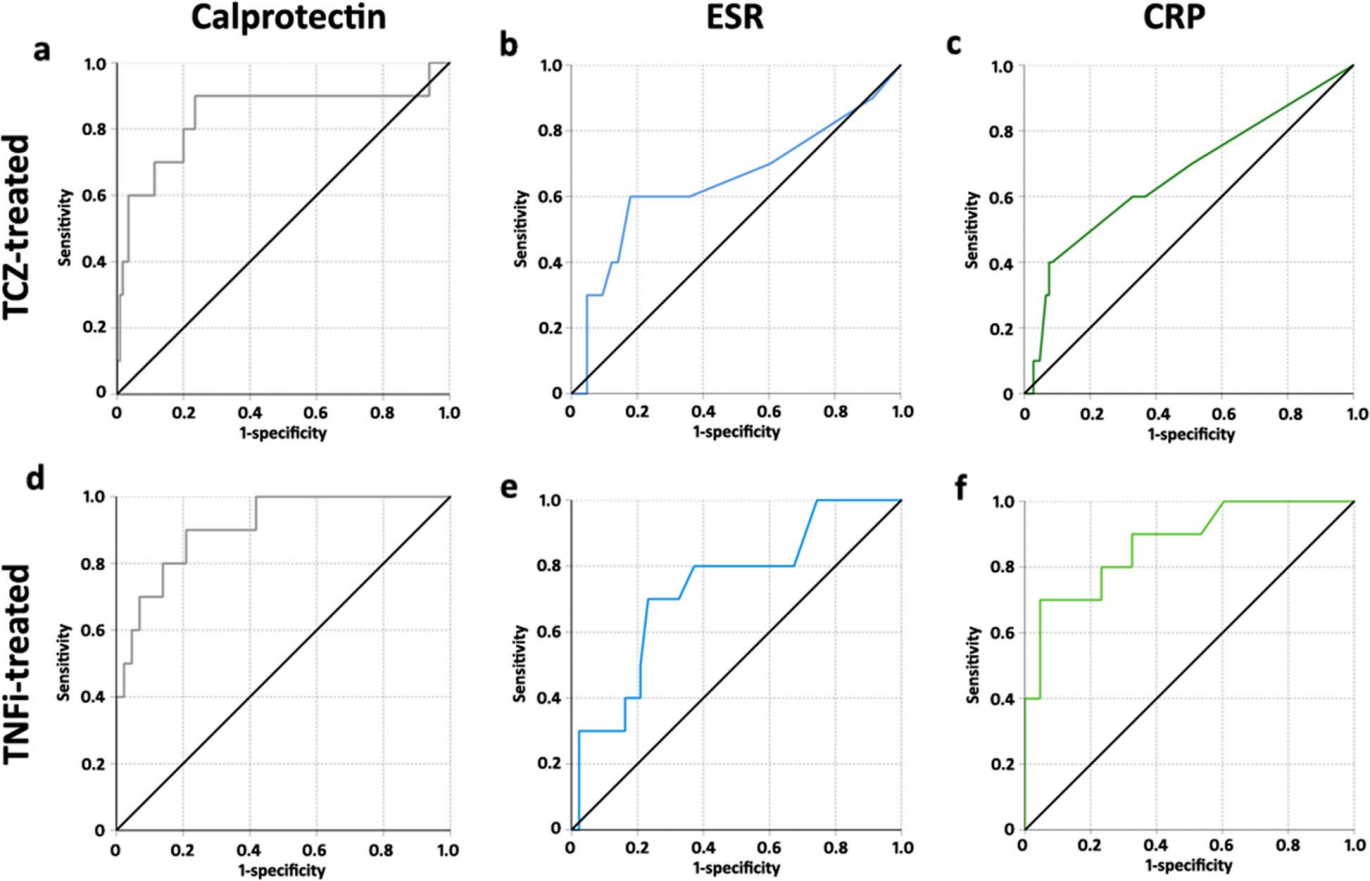
Receiver-operator characteristic (ROC) analyses to describe specificities and sensitivities in distinguishing active from not active RA of calprotectin (**a**, **d**), ESR (**b**, **e**), and CRP (**c**, **f**) in tocilizumab (TCZ)-treated RA patients (**a**–**c**) and in tumor necrosis factor alpha-inhibitor (TNFi)-treated (**d**–**f**) RA patients

To determine the calprotectin cut-off value with the best specificity and sensitivity, to detect RA activity requiring escalation or a change in DMARDs, ROC analysis was performed. A calprotectin value of 1916.5 ng/ml resulted in a sensitivity of 80.0% and a specificity of 80.0% (area under the curve [AUC] 0.841, 95 % CI 0.670–1.012, *P* < 0.001) (Fig. 2a).

#### Calprotectin correlates with CDAI and IL-6-dependent composite scores in tocilizumab-treated RA

**Fig. 3:**
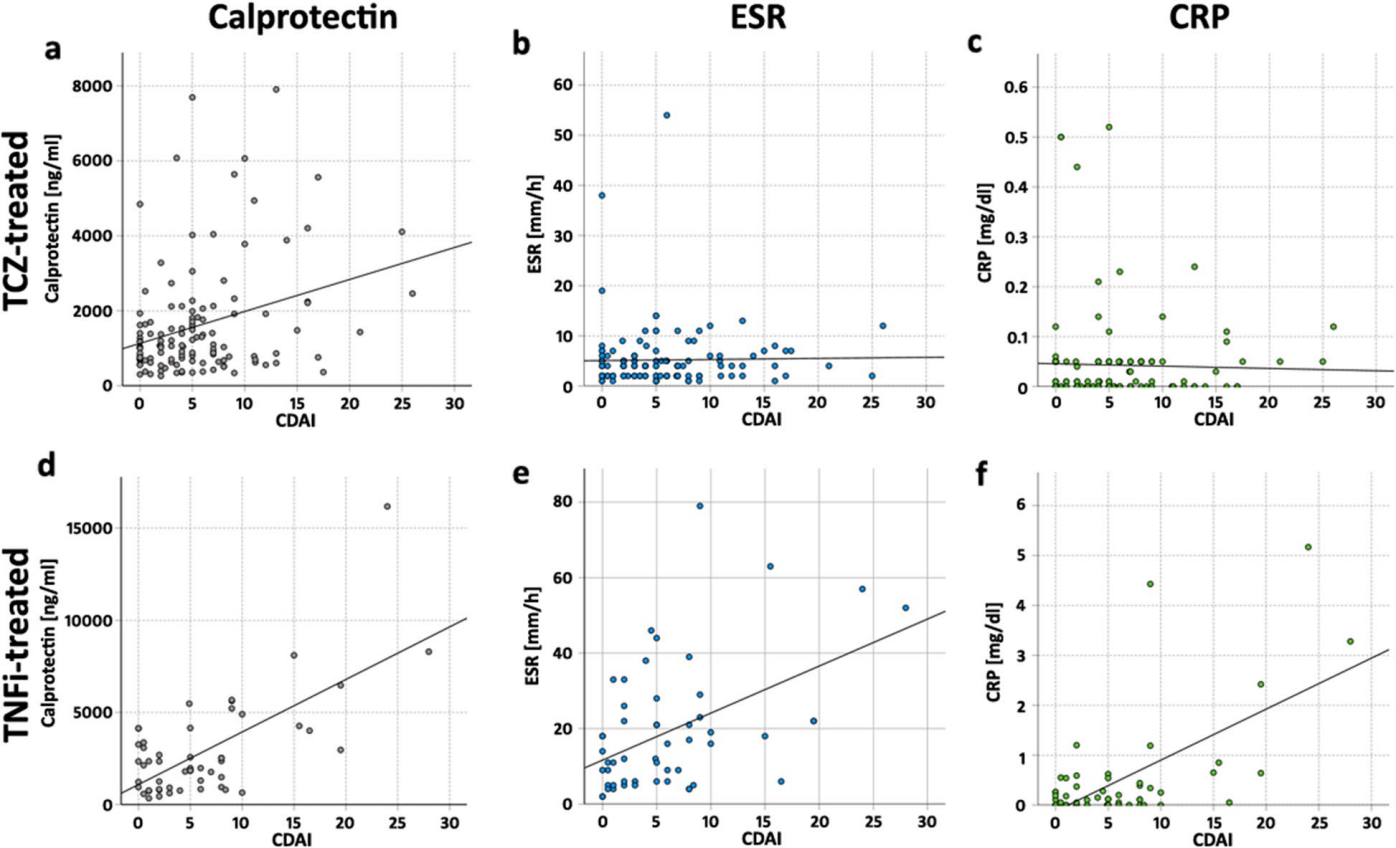
Scatterplots of calprotectin (**a**, **d**), ESR (**b**, **e**), and CRP values (**c**, **f**) with corresponding clinical disease activity index (CDAI) values in tocilizumab (TCZ)-treated RA patients (**a**–**c**) and in tumor necrosis factor alpha-inhibitor (TNFi)-treated (**d**–**f**) RA patients. Significant correlations were detected between calprotectin and CDAI in TCZ- (**a**) and TNFi-treated patients (**d**), as well as between ESR (**e**) and CRP (**f**) with CDAI in TNFi-treated patients

**Fig. 4:**
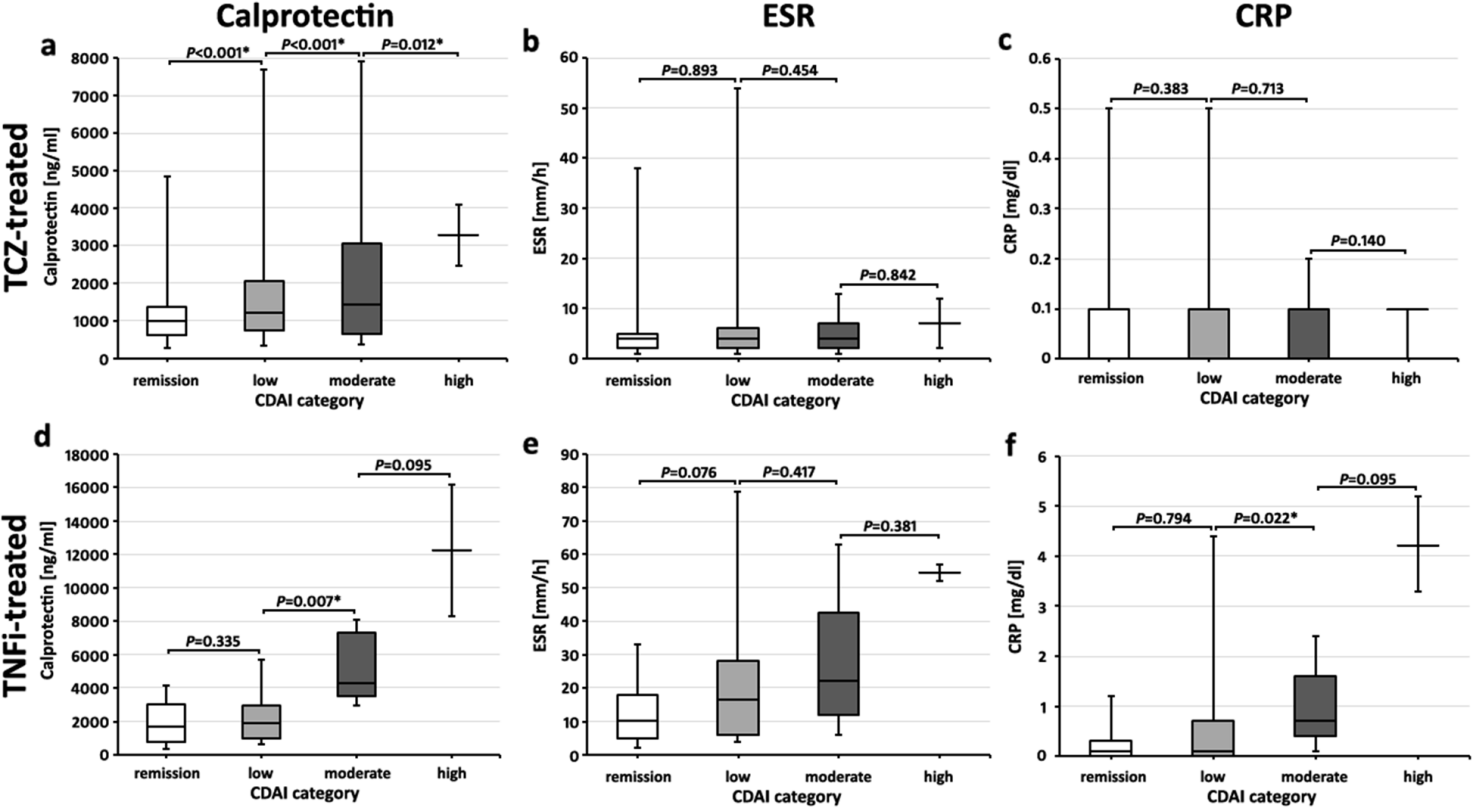
Boxplots of inflammation parameters with respective clinical disease activity index (CDAI) categories. Shown are calprotectin (**a**, **d**), ESR (**b**, **e**), and CRP values (**c**, **f**) in tocilizumab (TCZ)-treated RA patients (**a**–**c**) and in tumor necrosis factor alpha-inhibitor (TNFi)-treated RA (**d**–**f**) patients. Boxplots show medians with 25th and 75th percentiles; whiskers indicate minimums and maximums, respectively. *Significant differences in a Mann-Whitney *U* test

Calprotectin values showed a weak but significant correlation with CDAI values (*r* = 0.228; *P* = 0.011) (Fig. 3a) and significant differences of the median calprotectin values between the CDAI categories: Patients with CDAI-remission (*n* = 39) had a lower median calprotectin value compared to patients with CDAI-low activity (*n* = 67) (992.0 [615.0–1373.0] ng/ml vs 1225.0 [735.0–2059.0] ng/ml, *P* < 0.001). Patients with CDAI-low activity had lower median calprotectin levels than patients with CDAI-moderate activity (*n* = 17) (1430.0 [657.5–3061.5] ng/ml, *P* < 0.001). Patients with CDAI-moderate disease activity had lower median calprotectin levels than patients with CDAI-high activity (*n* = 2) (3283.0 ng/ml, *P* = 0.012) (Fig. 4a).

Additionally, a significant correlation of calprotectin (despite TCZ-treatment) with IL-6-dependent composite scores was detected: with DAS28-ESR (*r* = 0.301; *P* < 0.001), with DAS28-CRP (*r* = 0.237; *P* = 0.008), and with SDAI (*r* = 0.234; *P* = 0.009) (Supplemental Figure S1).

#### Standard inflammation parameters are not helpful to detect disease activity during tocilizumab treatment

ESR in 1h and CRP were evaluated in the TCZ-treated RA patients. No differences could be detected in the two parameters between not active patients compared to active patients (ESR: 4.0 [2.0–5.0] mm vs 7.0 [2.0–12.0] mm, *P* = 0.104, Fig. 1b. CRP 0.0 [0.0–0.1] mg/dl vs 0.1 [0.0–0.1] mg/dl, *P* = 0.075, Fig. 1c).

ESR and CRP had low sensitivities and specificities in TCZ-treated RA patients to distinguish active from not active RA patients (Fig. 2b and c, respectively).

No significant correlations could be detected between ESR (*r* = 0.010, *P* = 0.914, Fig. 3b) or CRP (*r* = −0.071, *P* = 0.432, Fig. 3c) with CDAI values.

ESR and CRP median values were not different between the CDAI-categories (ESR: CDAI-remission 4.0 [2.0–5.0] mm vs CDAI-low activity 4.0 [2.0–6.0] mm, *P* = 0.893; CDAI-moderate activity 4.0 [2.0–7.0] mm, *P* = 0.454; CDAI high activity 7.0 mm, *P* = 0.842, Fig. 4b. CRP: CDAI-remission 0.0 [0.0–0.1] mg/dl vs CDAI-low activity 0.0 [0.0–0.1] mg/dl, *P* = 0.383; CDAI-moderate activity 0.0 [0.0–0.1] mg/dl, *P* = 0.713; CDAI high activity 0.1 mg/dl, *P* = 0.140, Fig. 4c).

### RA patients receiving TNFα-inhibitors

#### Characteristics of RA patients receiving TNFα-inhibitors

Forty-five RA patients taking TNFα-inhibitors (TNFi, for at least 3 months) were included in this study. Patients’ characteristics including sex, age, RF/ACPA-status, concomitant DMARDs, median activity scores, and median inflammation parameters are embedded in Table 1. The comparison of RA patients taking TCZ with TNFi showed that patients taking TNFi had more often concomitant methotrexate medication and less often biological monotherapy. Patients taking TCZ had lower DAS28-ESR and DAS28-CRP scores, compared to patients with TNFi, whereas no differences were seen in SDAI and CDAI scores. Median ESR, CRP, and calprotectin values were lower in the TCZ group (Table 1).

#### Calprotectin in TNFα-inhibitor-treated active RA

Fifty-three calprotectin values were included in this study as from some patients more than one time point was measured. Not active RA patients receiving TNFi treatment (*n* = 43) had a lower serum-calprotectin (1845.0 [832.0–2569.0] ng/ml) than active RA patients (*n* = 10) receiving a change of their TNFi due to inflammatory activity of RA (5422.0 [3749.0–8150.8] ng/ml, *P* < 0.001) (Fig. 1d).

#### Sensitivity and specificity of calprotectin in detection of RA activity in TNFα-inhibitor-treated RA patients

The calprotectin cut-off value with the best specificity and sensitivity was calculated with ROC analysis. A calprotectin value of 3690.5 ng/ml resulted in a sensitivity of 80.0% and a specificity of 86.0% (area under the curve [AUC] 0.909, 95 % CI 0.817–1.000, *P* < 0.001) (Fig. 2d).

#### Calprotectin correlates with CDAI and IL-6-dependent composite scores in TNFα-inhibitor-treated RA patients

Calprotectin values correlated with CDAI values (*r* = 0.401; *P* = 0.001) (Fig. 3d). Patients with a CDAI-remission (*n* = 20) had a median calprotectin value of 1694.0 [773.5–2983.0] ng/ml, which was not significantly different from patients with CDAI-low activity (*n* = 26) (1848.0 [937.8–2965.5] ng/ml, *P* = 0.335). Patients with CDAI-low activity had lower median calprotectin levels than patients with CDAI-moderate activity (*n* = 5) (4264.0 [3488.0–7286.5] ng/ml, *P* = 0.007). Patients with CDAI-moderate disease activity had numerically but not significantly lower median calprotectin levels than patients with CDAI-high activity (*n* = 2) (12239.5 ng/ml, *P* = 0.095) (Fig. 4d). A significant correlation of calprotectin with IL-6-dependent composite scores was also present: with DAS28-ESR (*r* = 0.337; *P* = 0.005), with DAS28-CRP (*r* = 0.420; *P* = 0.002), and with SDAI (r = 0.412; *P* = 0.002) (Supplemental Figure S1).

#### Standard inflammation parameters in TNFα-inhibitor-treated RA

In contrast to TCZ-treated patients, in TNFi-treated RA patients ESR and CRP showed differences between not active and active patients (ESR: 12.0 [5.0–21.0] mm vs 22.5 [15.0–53.3] mm, *P* = 0.021, Fig. 1e. CRP: 0.1 [0.0–0.3] mg/dl vs 0.8 [0.3–0.3.6] mg/dl, *P* < 0.001, Fig. 1f).

ESR and CRP had higher sensitivities and specificities in TNFi-treated RA patients to distinguish active from not active RA patients compared to TCZ-treated RA patients (Fig. 2e and f).

Different from TCZ-treated patients, in TNFi-treated patients, significant correlations between CDAI and ESR (*r* = 0.410, *P* = 0.002, Fig. 3e) or CRP (*r* = 0.364, *P* = 0.007, Fig. 3f) were detected.

Median values of ESR in 1h were not significantly different between the CDAI-categories (ESR in CDAI-remission 10.0 [5.0–18.0] mm vs ESR in CDAI-low activity 16.5.0 [6.0–28.3.0] mm, *P* = 0.076; ESR in CDAI-moderate activity 22.0 [12.0–42.5] mm, *P* = 0.417 vs low CDAI activity; CDAI-high activity median 54.5 mm, *P* = 0.381) (Fig. 4e). Median CRP values between the CDAI categories were analyzed: CRP in CDAI-remission 0.1 [0.0-0.3] mg/dl vs CRP in CDAI-low activity 0.1 [0.0–0.4] mg/dl, *P* = 0.794; CRP in CDAI-moderate activity 0.7 [0.4–1.6] mg/dl, *P* = 0.022 vs CDAI-low activity; median CRP in CDAI high activity 4.2 mg/dl, *P* = 0.095 vs CRP in CDAI-moderate activity) (Fig. 4f).

## Discussion

In this study, calprotectin was measured in the serum of 114 patients with RA, of whom 69 were treated with TCZ and 45 with TNFi. The value of calprotectin as inflammation parameter, despite IL-6-receptor inhibition by TCZ, was evaluated. It could be demonstrated that calprotectin in the serum is suitable to detect RA activity despite TCZ treatment with good sensitivity and specificity of 80%. Furthermore, a significant correlation between the clinical composite score CDAI and calprotectin despite TCZ-treatment was found. In contrast, standard inflammation parameters, comprising ESR and CRP, could not detect activity in TCZ-treated RA patients.

Only one other group investigated calprotectin in the serum of TCZ-treated RA-patients by enzyme-linked immune-assay (ELISA) and also found significant correlations between calprotectin values and RA-activity composite scores [15], which is in concordance with our findings.

Disease activity scores, including DAS28-ESR and DAS28-CRP, which contain serological inflammation parameters for their calculation (to a relevant extent), were lower in our TCZ-treated group than in the TNFi-group. In prior studies, RA patients receiving TCZ-treatment had higher DAS28-ESR remission rates than CDAI or SDAI remission rates, which was supposed to be due to the high weight of the ESR in the DAS28-ESR score [3]. Therefore, our approach was to choose the CDAI as activity score for RA, due to CDAI’s independence of IL-6-influenced inflammation parameters. This approach was supported by the finding, that the CDAI was not different between our TCZ- and TNFi-treated RA patients. The SDAI was also not different between the two groups, although the score includes the CRP value. It already has been demonstrated that the CRP value influences the SDAI only to a low extent [3, 23]. ESR and CRP did not correlate with CDAI values in TCZ-treated RA patients, but a correlation of these two parameters with CDAI values could be seen in TNFi-treated patients. This finding could mean that standard inflammation parameters are less suitable to detect inflammatory activity, than calprotectin, as calprotectin correlated with CDAI values despite TCZ-treatment. Nevertheless, single parameters are less powerful to evaluate disease activity than composite scores [24]. An implementation of inflammation parameters, which are little disturbed by IL-6 receptor inhibition, into composite scores has to be considered, when IL-6-blockage is used.

It was formerly shown that RA patients with detectable TCZ trough levels (after intravenous application) did not have lower calprotectin values than patients with undetectable TCZ trough levels, in contrast to the CRP values, which were lower when TCZ was detectable [15]. This suggests calprotectin to be unaffected by TCZ. But in our study calprotectin appeared not to be fully independent of IL-6-receptor inhibition by TCZ as the median calprotectin values were lower in TCZ-treated patients than in TNFi-treated patients. The cut-off value of calprotectin for detecting active RA from not active RA was 1916.5 ng/ml with TCZ treatment and 3690.5 ng/ml with TNFi treatment. Calprotectin thereby exhibited comparable sensitivities and specificities between TCZ and TNFi treatment. A higher RA activity in TNFi-treated patients seemed not to be the reason for the differing median calprotectin value, as median CDAI values were not different between TCZ-treated and TNFi-treated patients.

The CDAI score is usually assessed directly after each patient’s visit without having the inflammatory parameters available at that time. This was also the case in our study: The CDAI scores were assessed without knowing the respective calprotectin values (neither ESR nor CRP). Thus, the decision of the investigator on the CDAI score was not biased by the knowledge of the inflammatory parameters.

Concerns over the pre-analytical factors of serum-calprotectin values have been raised, but recently it was demonstrated that serum-calprotectin is stable for 1 week when kept refrigerated [22]. This simple handling offers the possibility to transfer serum-calprotectin measurements into routine analysis.

Our study is limited due to its retrospective design and single-center approach. Additionally, future longitudinal studies are needed to evaluate calprotectin as follow-up parameter and its usefulness in detecting treatment response. Most of our patients had a low disease activity with a median CDAI value of 5, so that high inflammation parameters were only included in our study to a limited extent.

## Supplementary Information

**Additional file 1: Supplemental Figure S1** Correlation of interleukin-6-dependend composite scores including DAS28-ESR (a, d), DAS28-CRP (b, e) and SDAI (c, f) with calprotectin in tocilizumab (TCZ)-treated RA patients (a-c) and in tumor necrosis factor alpha-inhibitor (TNFi)-treated RA (d-f) patients.

## Data Availability

The data used and/or analyzed during the current study are available from the corresponding author on reasonable request.

## Abbreviations

ACPA: Anti-citrullinated protein antibody
ACR: American College of Rheumatology
AUC: Area under the curve
CDAI: Clinical disease activity index
CRP: C-reactive protein
DAS: Disease activity score
DMARD: Disease-modifying anti-rheumatic drug
EULAR: The European League Against Rheumatism
ESR: Erythrocyte sedimentation rate
IL: Interleukin
IQR: Interquartile range
LEF: Leflunomide
MTX: Methotrexate
na: Not applicable
RA: Rheumatoid arthritis
RF: Rheumatoid factor
ROC: Receiver operating characteristic
SDAI: Simplified disease activity index
TCZ: Tocilizumab
TNFi: Tumor necrosis factor alpha-inhibitor
