# Supplementary figures and images for: Calprotectin (S100A8/S100A9) detects inflammatory activity in rheumatoid arthritis patients receiving tocilizumab therapy
Source: Arthritis Res Ther. 2022 Aug 19;24:200. doi: 10.1186/s13075-022-02887-7 (PMC9389811; doi:10.1186/s13075-022-02887-7)

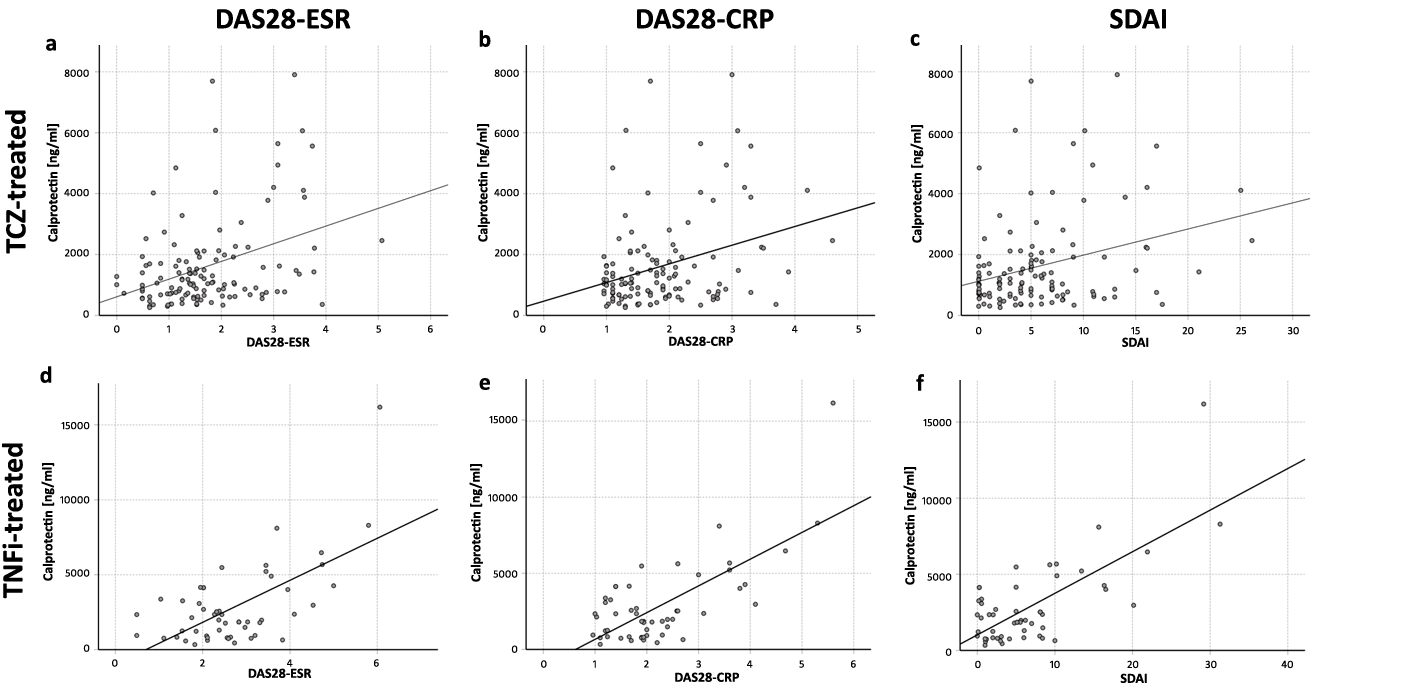

Supplement: Supplementary file 1 — Additional file 1: Supplemental Figure S1 Correlation of interleukin-6-dependend composite scores including DAS28-ESR (a, d), DAS28-CRP (b, e) and SDAI (c, f) with calprotectin in tocilizumab (TCZ)-treated RA patients (a-c) and in tumor necrosis factor alpha-inhibitor (TNFi)-treated RA (d-f) patients. [file 13075_2022_2887_MOESM1_ESM.tif]
